# Complex of MUC1, CIN85 and Cbl in Colon Cancer Progression and Metastasis

**DOI:** 10.3390/cancers7010342

**Published:** 2015-02-10

**Authors:** Sandra Cascio, Olivera J. Finn

**Affiliations:** 1 Department of Immunology, University of Pittsburgh School of Medicine, E1040 Biomedical Science Tower, Pittsburgh, PA 15261, USA; 2 Fondazione Ri.Med, via Bandiera, Palermo 90133, Italy

**Keywords:** MUC1/CIN85/Cbl complex, protein-protein interaction, lymph node metastasis

## Abstract

We previously reported that CIN85, an 85 KDa protein known to be involved in tumor cell migration and metastasis through its interaction with Cbl, associates with MUC1 in tumor cells. MUC1/CIN85 complex also regulates migration and invasion of tumor cells *in vitro*. Here, we examined specifically human colon carcinoma tissue microarrays (TMA) by immunohistochemistry for the expression of MUC1 and CIN85 and their potential role in cancer progression and metastasis. We detected a significant increase in expression of both MUC1 and CIN85 associated with advanced tumor stage and lymph node metastasis. We further investigated if Cbl could also be present in the MUC1/CIN85 complex. Co-immunoprecipitation assay showed that Cbl co-localized both with CIN85 and with MUC1 in a human colon cancer cell line. To begin to investigate the *in vivo* relevance of MUC1 overexpression and association with CIN85 and Cbl in cancer development and progression, we used human MUC1 transgenic mice that express MUC1 on the colonic epithelial cells, treated with azoxymethane to initiate and dextran sulfate sodium (AOM/DSS) to promote colorectal carcinogenesis. MUC1.Tg mice showed higher tumor incidence and decreased survival when compared with wild-type mice. Consistent with the *in vitro* data, the association of MUC1, CIN85 and Cbl was detected in colon tissues of AOM/DSS-treated MUC1 transgenic mice. MUC1/CIN85/Cbl complex appears to contribute to promotion and progression of colon cancer and thus increased expression of MUC1, CIN85 and Cbl in early stage colon cancer might be predictive of poor prognosis.

## 1. Introduction

Mucin 1 (MUC1) is a transmembrane glycoprotein expressed primarily on apical surfaces of ductal epithelia. MUC1 structure includes the *N*-terminal extracellular domain (MUC1-N) non-covalently linked to the smaller subunit carrying the transmembrane domain (TM) and the cytoplasmic tail (CT). The extracellular domain of MUC1 contains a variable number of tandem repeats (VNTR) composed of between 20 to more than 200 tandem repeat (TR) units of 20 residues each, rich in prolines and *O*-glycosylated on serines and threonines. On normal epithelia, MUC1 is heavily glycosylated and expressed at low levels, while it is overexpressed and markedly hypoglycosylated in epithelial cancers such as colon, pancreas, breast, prostate and bladder, where it confers poor prognosis [1,2]. Elevated levels of the hypoglycosylated MUC1 in tumors have been associated with increased invasiveness and metastasis of tumor cells [3,4,5,6].

Previous studies showed that the MUC1 cytoplasmic tail (MUC1.CT) domain is involved in a wide range of intracellular signaling by association with numerous kinases, cell adhesion molecules, transcription factors and chaperones that are implicated in malignant transformation [7,8]. Recently we published that the extracellular domain and the VNTR also participate in intracellular signaling by activating NF-κB pathway [9] and regulating invasiveness and metastasis processes in a complex with CIN85, a Cbl-interacting protein of 85 kDa [3]. In particular, we showed that underglycosylated form of MUC1 and CIN85 co-localize on invadopodia structures regulating migration and invasion of breast cancer cells. These data suggested that overexpression of CIN85 and MUC1 in tumors could promote malignancy by increasing invasiveness of cancer cells [3].

CIN85 is a multifunctional adaptor/scaffold protein composed of three *N*-terminal SH3 domains followed by a proline-rich region and a *C*-terminal coiled-coil region [10]. In association with c-Cbl (Casitas B-lineage lymphoma), an E3 ubiquitin ligase, CIN85 controls the intracellular internalization, trafficking and sorting of several activated receptor tyrosine kinases (RTK), including the epidermal growth factor receptor (EGFR). Moreover, through the SH3 domains and the proline-rich region, CIN85 is implicated in many protein-protein interactions and it is found to play important roles in other processes such as apoptosis, rearrangement of actin cytoskeleton, cell adhesion, immunological synapse, cell migration and invasion [10,11]. However, it appears that CIN85 must associate with Cbl in order to regulate intracellular signaling such as the antigen receptor signaling in human B cells [12], interaction and modulation of TRAIL-induced MEKK4/p38/Akt survival network [13], and mono-ubiquitination of AMAP1 to drive invasion of breast cancer cells [14].

Being that we had shown the importance of the MUC1/CIN85 complex in tumor cells, we questioned if the activities of that complex were also dependent on the presence of Cbl. We therefore investigated the potential association of the tumor form of MUC1 and CIN85 with Cbl in colon cancer cells *in vitro* and during colon cancer development *in vivo*. Our data suggest that an increase in the expression of these molecules and their co-association might play a critical role in the promotion and progression of colon and most likely other cancers.

## 2. Results

### 2.1. Detection of CIN85 and Abnormal MUC1 in Human Colon Cancer

We have previously shown that the tumor form of MUC1 and CIN85 are expressed at significantly higher levels in early and advanced stage of human breast cancer compared with normal epithelium [3]. Here, we analyzed expression of abnormal MUC1 and CIN85 in human colon cancer tissues microarrays (TMA). The TMA contained tissues from four normal, 16 benign, including polyps and adenoma, and 55 malignant cases, with each sample in duplicate.

**Figure 1:**
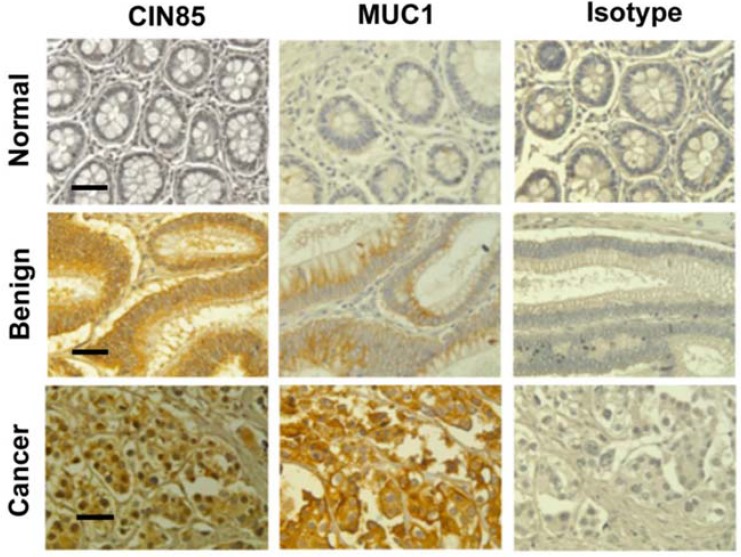
CIN85 and MUC1 are overexpressed in human colon adenocarcinomas. Immunohistochemistry staining for tumor form of MUC1 and CIN85 in normal, benign and cancer tissues. MUC1 staining was performed by using anti-MUC1 VU 4H5 antibody. Scale bar 100 μm.

In tissue samples from patients with benign tumors, the intensity of MUC1 VU4H5 staining was very weak or moderate (Table 1) whereas CIN85 staining displayed a moderate intensity. Specimens of adenocarcinoma samples revealed strong staining with both MUC1 VU4H5 and anti-CIN85 antibodies (Figure 1). In all tissue samples, abnormal MUC1 and CIN85 were localized both at the plasma membrane and in the cytosol.

**Table 1:** Expression of abnormal MUC1 and CIN85 in human colon tissues.

Characteristics	No.	MUC1 *				*P*-Value	CIN85				*P*-Value
		Neg	Weak	Moderate	High		Neg	Weak	Moderate	High	
**Normal**	4	3	1	0	0	0.048	2	2	0	0	0.082
**Benign**	16	5	6	5	0		3	3	8	3	
**Cancer**	55	9	15	14	14		5	12	19	19	

* Abnormal MUC1.

Next, we investigated the correlation of tumor form of MUC1 and CIN85 expression levels with lymph node positivity and metastasis. As indicated in Table 2, of total 31 samples positive for lymph node, 19 (60%) showed moderate and high expression of abnormal MUC1 (MUC1 ^Mod/High^) and 23 (74%) had moderate and high expression of CIN85 (CIN85 ^Mod/High^). Significantly, MUC1 and CIN85 were found to be co-expressed in 17 (54%) tumors with lymph node positivity. Co-expression of abnormal MUC1 and CIN85 were also found significant in all distant metastatic tissues (Table 2). Both MUC1 and CIN85 showed strong staining of the entire tissue section.

**Table 2:** Co-expression of MUC1 and CIN85 in lymph node positivity and distant metastasis in colon cancer.

Group	No.	MUC1 ^* Mod/HIgh^ CIN85 ^Neg/Low^	MUC1 ^* Neg/Low^ CIN85 ^Mod/High^	MUC1 ^*^/CIN85 ^(Mod/High)^		*P*-Value
				Co-Expression	No Co-Expression	
LN Positive	31	2	6	17	6	0.043
LN Negative	24	6	9	6	3	
Metastasis Positive	6	0	0	6	0	0.024
Metastasis Negative	49	8	15	17	9	

* Abnormal MUC1.

### 2.2. Novel Association of Cbl with CIN85 and MUC1

**Figure 2:**
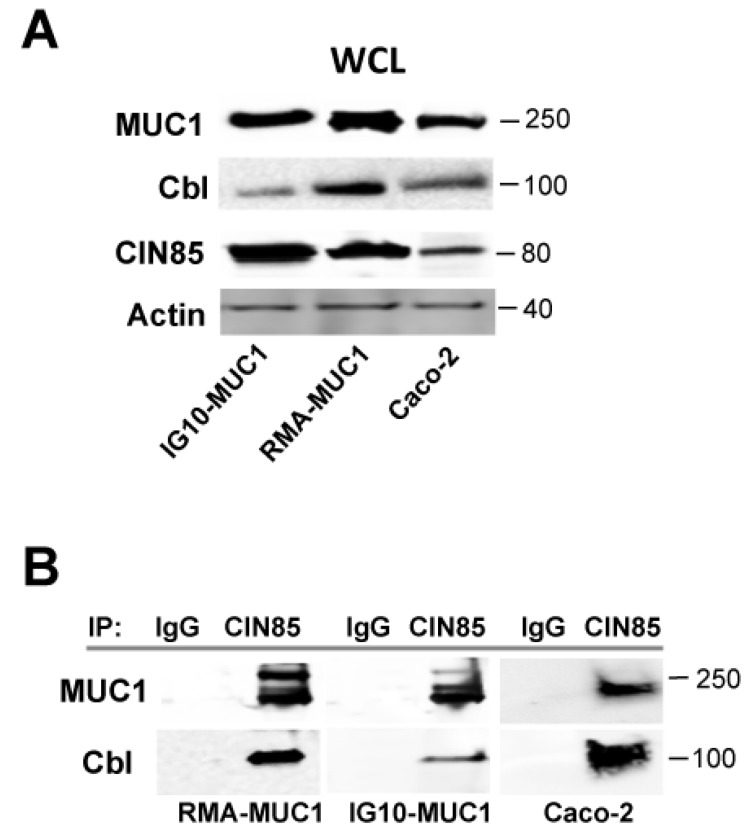
MUC1 and CIN85 complex contains Cbl. (**A**) Whole cell lysate (WCL) from the MUC1-transfected IG10 (IG10/MUC1) and RMA (RMA/MUC1) mouse tumor cell lines, and Caco-2 human colon cancer cell line was immunoblotted with anti-MUC1 antibody 4H5, anti-CIN85 and anti-Cbl antibodies. Immunoblotting with beta-actin antibody was carried out as a loading control; (**B**) SDS-PAGE of RMA/MUC1, IG10/MUC1 or Caco-2 cells lysates immunoprecipiated with anti-CIN85 antibody or control IgG and immunoblotted with anti-MUC1 VU 4H5 and anti-Cbl antibodies.

We have previously shown that the complex of CIN85 and MUC1 plays a crucial role in matrix degradation and invasion by different breast cancer cells. Association between CIN85 and MUC1 was clearly detected both in cell lines stably transfected with MUC1 and in cell lines that express endogenous MUC1 [3]. As CIN85 is a multi-adaptor protein and was previously shown to strongly bind to Cbl [15,16,17], we tested whether the newly discovered MUC1/CIN85 complex might also contain Cbl. We used two mouse cancer cell lines stably transfected with human MUC1 cDNA, the lymphoma cell line RMA (RMA-MUC1) and the mouse ovarian cancer cell line IG10 (IG-10/MUC1), as well as the human colon cancer cell line Caco-2 that spontaneously expresses MUC1. First, we analyzed individually the expression of human MUC1, CIN85 and Cbl by western blotting (Figure 2A).

Next, whole lysates were immunoprecipitated with anti-CIN85 antibody, or with a control mouse IgG, the precipitates run on an SDS gel and immunoblotted with anti-MUC1 VU 4H5 and anti-Cbl antibodies (Figure 2B). Confirming the previous observation of the association of CIN85 and MUC1, we saw abnormal MUC1, detected by anti-MUC1 antibody VU 4H5 specific for the hypoglycosylated VNTR, in the CIN85 immunoprecipitates from RMA/MUC1, IG-10/MUC1 and Caco-2. There was also a clear band corresponding to the molecular weight of Cbl detected with anti-Cbl antibody, indicating co-immunoprecipitation of all three molecules, CIN85, Cbl and MUC1.

### 2.3. MUC1, CIN85 and Cbl in AOM/DSS-Induced Mouse Colon Carcinogenesis

**Figure 3:**
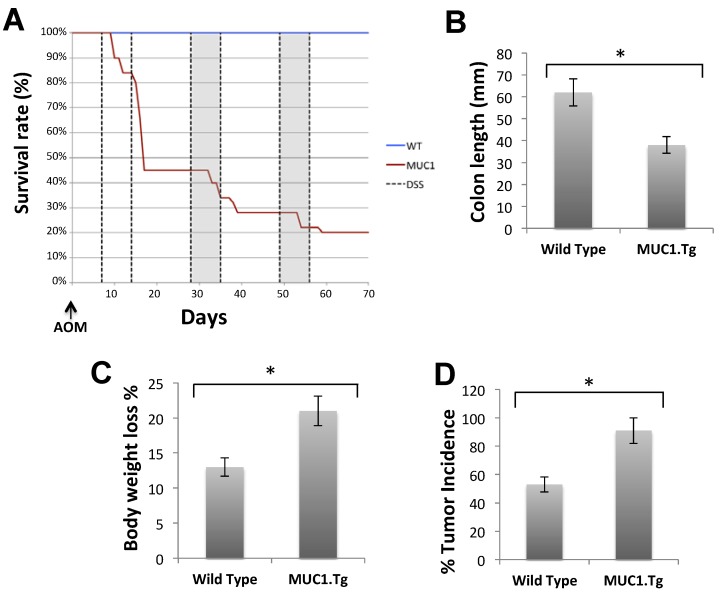
MUC1 enhances cancer progression in AOM/DSS-treated mice. WT and MUC1.Tg mice were given an AOM injection followed by three cycles of 1.5% DSS in drinking water, as described in Materials and Methods. (**A**) Kaplan-Meier survival curves of WT (*n* = 38) and MUC1.Tg (*n* = 40) during AOM/DSS treatment; (**B**) Measurement of colon length in sacrificed mice (*n* = 8); (**C**) Percentage of weight loss in mice at day 14 following one cycle of DSS; (**D**) Incidence of tumors in the colons.

We examined *in vivo* the expression of and association between abnormal MUC1, CIN85 and Cbl in human MUC1 transgenic mice subjected to AOM/DSS-induced colon carcinogenesis and compared with MUC1 negative WT mice. As shown in Figure 3, administration of AOM/DSS induced tumor incidence (Figure 3D) and classic colitis symptoms in all the mice. However, MUC1 transgenic (MUC1.Tg) mice showed a significantly decreased survival (Figure 3A), reduced colon length (Figure 3B) and higher loss of body weight (Figure 3C) compared to the wild-type (WT) mice. The length of colon and survival did not differ between WT and MUC1.Tg mice in the untreated control group (data not shown). MUC1.Tg mice also showed an increase in the incidence of colon tumors by 40% (Figure 3D). These data indicated that the presence of MUC1 accelerated AOM/DSS-induced carcinogenesis and progression to colon tumors.

**Figure 4:**
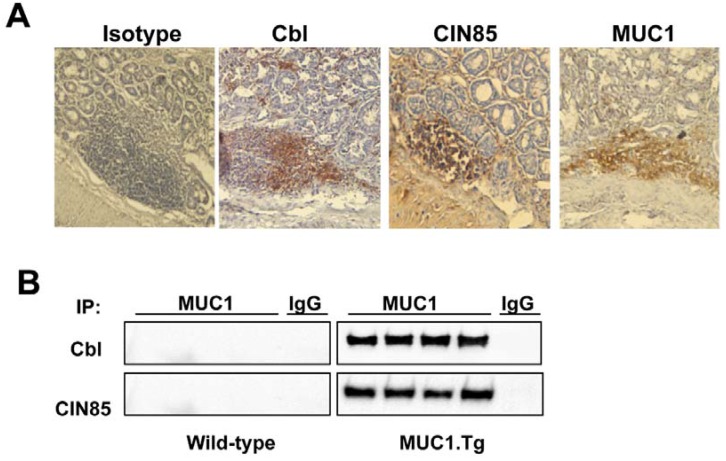
The presence of Cbl in colons of AOM/DSS-treated mice and *in vivo* association with the MUC1/CIN85 complex. (**A**) Immunohistochemistry of human MUC1.Tg mice colon tissues. Samples were fixed and stained with anti-human MUC1 antibody VU 4H5, anti-Cbl and anti-CIN85 antibodies; (**B**) Whole cell lysates (WCL) from colon tissues of AOM/DSS-treated human MUC1.Tg mice were immunoprecipitated with VU-4H5 antibody or control IgG and immunoblotted with anti-Cbl and anti-CIN85 antibodies.

We then evaluated the expression of human tumor MUC1 (detected by anti-MUC1 VU 4H5 antibody), CIN85 and Cbl in the colonic tissue of AOM/DSS-treated mice by immunohistochemistry (Figure 4A). MUC1.Tg mice showed high expression levels of all three proteins. Cell lysates from colon tissues were precipitated with anti-MUC1 VU-4H5 antibody and immunoprecipitated proteins run on SDS gel and immunoblotted with anti-CIN85 or anti-Cbl antibodies (Figure 4B). Immunoprecipiation with an unrelated IgG served as a negative control (Figure 4B) and immunoblotting with anti-actin antibody served as a control for equal loading of protein on the gel. As shown in Figure 4B, MUC1, CIN85 and Cbl co-precipitated in all MUC1.Tg mouse samples whereas no association was detected in WT mice.

## 3. Discussion

Tumor metastasis is a multistep process that includes migration, cell–cell adhesion, degradation of the surrounding matrix, intravasation to blood vessels and extravasation (invasion) into new tissues. Overexpression of MUC1 has been observed in many types of adenocarcinoma and correlated with lymph node metastasis and poor prognosis in patients [18,19,20,21]. Increased levels and altered glycosylation of MUC1 facilitate invasive growth and metastasis of tumor cells. MUC1 contributes to the invasive and metastatic properties of adenocarcinomas by mediating the epithelial to mesenchymal transition (EMT), the mechanism by which polarized epithelial cells acquire mesenchymal cell properties with an enhanced potential for migration, and modulating both adhesive and anti-adhesive properties of tumor cells [20]. MUC1, in association with other molecules such as β-catenin, NF-kB p65 or EGFR, regulates transcription of several genes responsible for progression and invasiveness of cancer [9,22,23]. Moreover, we recently reported the identification of CIN85 as a new partner of MUC1 and implicated the MUC1/CIN85 complex in invasion and metastasis of cancer cells. In the present study, we found MUC1 and CIN85 overexpressed in benign as well as malignant colon tumors with the highest co-expression levels observed in advanced clinical grades and primarily in colon adenocarcinomas with lymph node positivity and metastases.

Given that CIN85 had previously been reported to associate with Cbl, which was shown to be necessary for the CIN85-mediated regulation of intracellular signaling, we examined whether Cbl might also be in the MUC1/CIN85 complex in tumor cells. Here we report that Cbl is the third protein in the CIN85/MUC1 complex. Co-immunoprecipiation assay revealed that the extracellular domain of tumor form of MUC1 interacts with CIN85 and Cbl molecules in the plasma membrane and cytosol compartment of MUC1-transfected cells and also in colon cancer cells that normally express MUC1.

To examine *in vivo* the significance of MUC1 expression and its association with CIN85 and Cbl in colon cancer development and progression, we used the AOM/DSS mouse model of colorectal carcinogenesis. In particular, we analyzed the expression of CIN85 and Cbl in AOM-DSS-treated MUC1.tg and WT mice. Our results showed that CIN85 and Cbl are highly expressed in the affected colon tissues of treated human MUC1.Tg mice in association with MUC1.

Thus, our study finds that MUC1, CIN85 and Cbl are overexpressed and co-localize in colon cancer cells. Although the exact mechanism by which MUC1/CIN85/Cbl complex promotes and regulates cancer progression is still under investigation, this association is highly related to advanced stage of tumors and lymph node metastasis. Identification of CIN85 and Cbl as binding proteins of MUC1 provides the mechanistic support for its functions as the promoter of cancer invasiveness and metastasis and suggests that drugs capable of breaking this complex might be effective at reducing metastatic potential of tumors.

## 4. Experimental Section

### 4.1. Cell Culture

RMA mouse T cell lymphoma and IG-10 mouse epithelial tumor cell lines were grown in DMEM (Dulbecco’s Modified Eagle Medium, Cellgro, Mediatech, Inc., Herndon, VA, USA) whereas Caco-2 human cancer cells were grown in RPMI-1640 (Royal Park Memorial Institute Medium, Cellgro) medium. Both DMEM and RPMI were supplemented with 10% heat-inactivated fetal bovine serum, 100 units/mL penicillin, 100 μg/mL streptomycin and 2 mmol/L L-glutamine.

### 4.2. Plasmid and Transfection

We utilized the pcDNA3 core plasmid (Invitrogen Life Technology, Carlsbad, CA, USA), into which we inserted human MUC1 cDNA containing 22 tandem repeats. Transfection was performed with Lipofectamine™ 2000 (Invitrogen) according to the manufacturer’s instructions. Stable expression was achieved by selection in up to 1000 μg/mL G418 (Sigma-Aldrich, St Louis, MO, USA).

### 4.3. Animals and tumor induction

C57BL/6 mice were purchased from the Jackson Laboratory (Bar Harbor, ME, USA). MUC1 transgenic mice were originally purchased from Dr. S.J. Gendler (The Mayo Clinic, Scottsdale, AZ, USA) and are bred at the University of Pittsburgh (Pittsburgh, PA, USA). To induce colitis-associated cancer mice were i.p. injected with 10 mg/kg AOM and kept on regular water for 7 days. After 7 days mice received three 5-day cycles of 1.5% DSS (M.W. = 36,000–50,000; MP Biomedicals, Solon, OH, USA) in sterilized drinking water, followed by 14 days of sterilized tap water. All experiments were approved by the Institutional Animal Care and Use Committee of the University of Pittsburgh.

### 4.4. Western Blotting and Immunoprecipitation

Total cell proteins were extracted using RIPA buffer (150 mM NaCl, 0.5 sodium deoxycholate, 0.1% SDS, 1% NP-40 and 50 mM Tris-HCl) with commercial protease inhibitors (Complete Protease Inhibitor Cocktail from Roche, Mannheim, Germany) and phosphatase inhibitors (Phosphatase Inhibitor Cocktail II, Sigma-Aldrich, St Louis, MO, USA). Protein from lysates (50 μg) of was subjected to SDS-PAGE for western blotting (WB), while 500 μg was used for immunoprecipitation (IP). The following antibodies were employed: anti-CIN85 (A-7), anti-actin, anti-MUC1 4H5 (Santa Cruz Biotechnology, Santa Cruz, CA, USA); anti-Cbl (Abcam, Cambridge, MA, USA). For immunoprecipitation, protein lysates were first pre-cleared with protein G-Sepharose beads (Sigma-Aldrich) for 2 h at 4 °C, then incubated with primary antibody at 4 °C for 16 h. The immune complexes were precipitated for 2 h at 4 °C with Protein-G Sepharose 4B. In control samples, the primary Ab was substituted with non-immune mouse IgG (rabbit or mouse, depending on the source of the primary Abs). Immunoprecipitates were washed 3 times with RIPA lyses buffer containing 0.5 M NaCl, 2% sodium dodecyl sulfate (SDS), 3 times with RIPA lyses buffer and once with PBS, then were resuspended in Laemmli Buffer. The proteins were separated on a pre-cast 4%–20% polyacrylamide gel (BioRad, Hercules, CA, USA) immunoblotted, and immunoblots were developed with horseradish peroxidase-conjugated secondary antibody and chemoluminescence reagents (Thermo Fisher Scientific, Rockford, IL, USA). Intensity of signals was determined by Image J.

### 4.5. Immunohistochemistry

Human colon cancer tissue paraffin sections (catalog number Z7020036) were purchased from Biochain (Newark, CA, USA). The size of cores of TMA was 1.5 mm, cut 4 μm. Slides were deparaffinized by baking overnight at 59 °C. Endogenous peroxidase activity was eliminated by treatment with 30% H_2_O_2_ for 15 min at room temperature. Antigen retrieval was performed by microwave heating in 0.1% citrate buffer. Nonspecific binding sites were blocked with Protein Blocking Agent (Thermo Fisher Scientific). The anti-MUC1 Ab 4H5, which recognizes the epitope APDTRPAP in the VNTR region of the hypoglycosylated MUC1, was purchased from Santa Cruz Biotechnology. The anti-CIN85 Ab (anti-SH3KBP1) was purchased from Novus Biologicals (Littleton, CO, USA) and anti-Cbl from Abcam. Staining was performed by the avidin-biotin-peroxidase complex method with a commercial kit (Vectastain ABC kit; Vector Laboratories, Burlingame, CA, USA). Positive signals were visualized by a DAB Kit (BD Pharmingen, San Jose, CA, USA).

### 4.6. Scoring of Immunoreactivity

Expression of MUC1 and CIN85 in the human normal and tumor tissues was scored as negative or (heterogeneously) positive. Evaluation of MUC1 and CIN85 antibody staining patterns was carried out in a semiquantitative manner. The staining intensity was graded as negative, weak, moderate or high.

### 4.7. Statistical Analysis

The χ^2^ test was used to estimate the statistical significance of difference between MUC1 and CIN85 expression and clinicopathological characteristics. Survival curve was assessed by the Kaplan-Meier method. Differences in data of two conditions were analyzed by the student’s *t* test. In all cases, *p* < 0.05 was considered to indicate a statistically significant difference.
